# *Staphylococcus aureus* Isolated from the Oral Cavity: Phage Susceptibility in Relation to Antibiotic Resistance

**DOI:** 10.3390/antibiotics10111329

**Published:** 2021-10-31

**Authors:** Katarzyna Garbacz, Ewa Kwapisz, Lidia Piechowicz, Maria Wierzbowska

**Affiliations:** 1 Department of Oral Microbiology, Medical Faculty, Medical University of Gdansk, 80-204 Gdansk, Poland; ewa.kwapisz@gumed.edu.pl (E.K.); maria.wierzbowska@gumed.edu.pl (M.W.); 2 Department of Medical Microbiology, Medical Faculty, Medical University of Gdansk, 80-204 Gdansk, Poland; lidia.piechowicz@gumed.edu.pl

**Keywords:** bacteriophage, phage therapy, *Staphylococcus aureus*, oral cavity, antibiotic resistance, MRSA, methicillin, PVL

## Abstract

Nowadays, research on bacteriophage therapy and its potential use in combination with antibiotics has been gaining momentum. One hundred and ten oral *Staphylococcus aureus* isolates were phage-typed and their antibiotic resistance was determined by standard and molecular methods. The prevalence of MSSA and MRSA strains was 89.1% and 10.9%, respectively. Nearly all (91.8%) analyzed isolates, whether MSSA or MRSA, were susceptible to the phages used from the international set. The highest lytic activity showed phages 79 and 52 A from lytic group I. The predominant phage groups were mixed, the I+III group and a mixed group containing phages from at least three various lytic groups. *S. aureus* strains sensitive to phage group I were usually resistant to penicillin and susceptible to ciprofloxacin, whereas the strains typeable with group V or group V with the 95 phage were susceptible to most antibiotics. Epidemic CA-MRSA strains (SCC*mec*IV) of phage type 80/81 carried Panton–Valentine leucocidin genes. Considering the high sensitivity of oral *S. aureus* to the analyzed phages and the promising results of phage therapies reported by other authors, phage cocktails or phage-antibiotic combinations may potentially find applications in both the prevention and eradication of staphylococcal infections.

## 1. Introduction

The phenomenon of bacterial resistance to antimicrobial agents is currently one of the most emerging global public health problems, leading to treatment failures in many patient groups [1]. *Staphylococcus aureus*, considered one of the most significant etiological factors of infection, developed multiple mechanisms of antibiotic resistance, which are transferred rapidly between the strains in both hospital and community settings [1]. The problem is particularly evident in the case of methicillin-resistant *S. aureus* (MRSA), which previously spread primarily in a hospital setting as hospital-acquired MRSA (HA-MRSA) but is nowadays increasingly found in community settings as community-acquired MRSA (CA-MRSA), displaying high infectivity and virulence [2,3,4].

The ineffectiveness of previously used antibiotic therapies warrants research on alternative treatments, such as phage therapy [5]. Bacteriophages (phages) are viruses capable of infecting and replicating inside bacterial cells, which ultimately leads to the destruction of the latter. Since their discovery, phages has raised hopes as a therapeutic option, but research on phage therapies has slowed down due to the growth of the global antibiotic market [6]. However, the emerging problem of antibiotic resistance stimulated research on phage therapies in many countries; the US National Institute of Allergy and Infectious Diseases listed phage therapy as one of the seven strategies to overcome antibiotic resistance [7].

One of the prerequisites of successful phage therapy is the sensitivity of eradicated bacteria to bacteriophages. Recent studies have shown that, similar to antibiotic resistance, bacteria can also develop resistance to bacteriophages [7]. In such cases, bacteriophage cocktails or phage-antibiotic combinations seem to be effective therapeutic options [8,9,10]. Phage-antibiotic combinations appear as the most realistic alternative to inefficient antibiotic therapy. Some published case reports demonstrated that bacteria could be effectively eradicated by administering a bacteriophage cocktail combined with antibiotics [11]. However, more evidence from in vitro studies is needed to optimize the synergism between these two therapies.

Our Polish Reference Center for Staphylococci is a leading center for bacteriophage typing of *S. aureus* strains involved in various infections, with considerable achievements in this field [12,13,14,15,16]. Recently, the oral cavity has gained attention as a reservoir of antibiotic-resistant *S. aureus* strains and a source of their spread to other anatomical regions [17,18,19]. However, the knowledge of the sensitivity of oral *S. aureus* to bacteriophages and the potential therapeutic application of the latter are fairly limited.

Considering the above, the aim of this study was to analyze the sensitivity of *S. aureus* isolated from the oral cavity to staphylococcal bacteriophages from the international phage typing set and to study the relationship between phage sensitivity and antibiotic resistance.

## 2. Results

### 2.1. Antibiotic Resistance of Oral S. Aureus Strains

The prevalence of MSSA (methicillin-sensitive *S. aureus*) and MRSA among 110 analyzed oral *S. aureus* isolates was 89.1% (*n* = 98) and 10.9% (*n* = 12), respectively. All MRSA strains harbored the methicillin resistance gene mecA and lacked the mecC gene. Type IV and V staphylococcal cassette chromosomes mec (SCCmec) were found in 66.7% and 33.3% of the isolates, respectively (Table 1).

**Figure 1:**
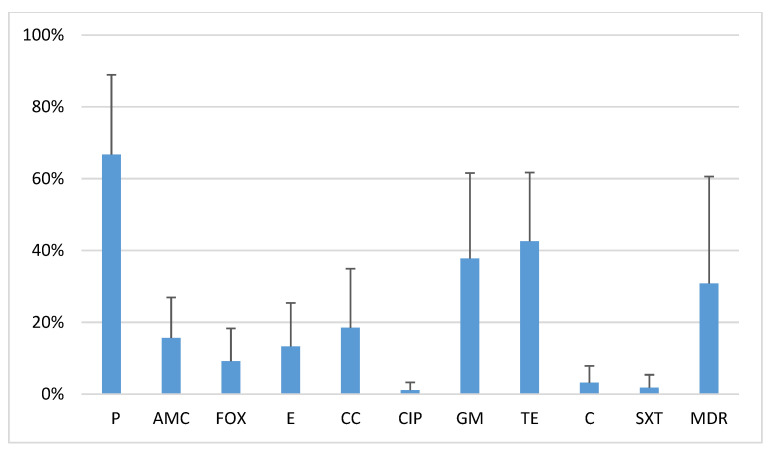
Antibiotic resistance of 110 oral *S. aureus* strains. P—penicillin; AMC—amoxicillin/clavulanic acid; FOX—cefoxitin; E—erythromycin; CC—clindamycin; CIP—ciprofloxacin; GM—gentamicin; TE—tetracycline; SXT—sulfamethoxazole/trimethoprim; MDR—multidrug resistance.

The study isolates showed various degrees of resistance to other antibiotics: penicillin G (63.6%), tetracycline (42.7%), gentamycin (30.9%), clindamycin (20%), erythromycin (18.2%), amoxicillin/clavulanic acid (18.2%), chloramphenicol (3.6%), trimethoprim/sulfamethoxazole (2.7%), and ciprofloxacin (1.8%). Multidrug-resistant (MDR) isolates constituted 29.1% of all strains (Figure 1).

### 2.2. Activity of Phages from Lytic Groups

**Figure 2:**
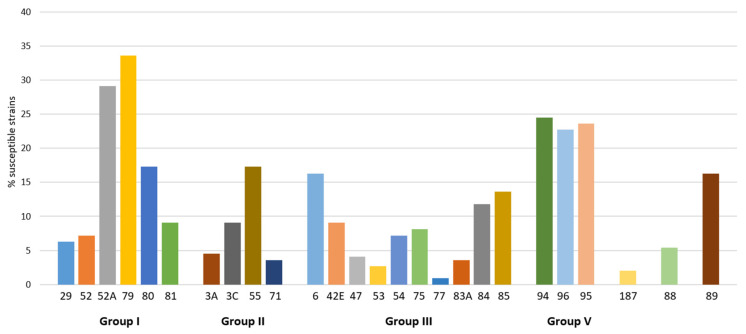
Sensitivity of 110 oral *S. aureus* strains to specific lytic phages from international set.

Nearly all (91.8%) analyzed *S. aureus* isolates turned out to be sensitive to selected phages from the international phage set. The highest lytic activity showed phages 79 and 52 A from lytic group I, with the proportions of sensitive strains of 30% and 27.3%, respectively. The second most active cluster consisted of phage 96 from group V and phage 95, with more than 20% of the strains sensitive to each. Other phages with proportions of sensitive strains higher than 15% were phage 80 (group I), phage 6 (group II), and, additionally, phage 89 (Figure 2).

The predominant phage groups were mixed, the I + III group and a mixed group containing phages from at least three various lytic groups, with percentages of sensitive strains of 18.2% and 17.3%, respectively. The proportions of isolates sensitive to phages from groups II, I, and V with type 95 were 16.4%, 15.5%, and 11.8%, respectively.

MRSA and MSSA strains differed in terms of their phage sensitivity, and MRSA strains were significantly more often sensitive to phages 80 and 81 from group I (50% vs. 12.2%) (*p* > 0.05) and phages 3C/71 from group II (25% vs. 15.3%) than MSSA strains were.

### 2.3. Activity of Phage Lytic Groups Versus Antibiotic Resistance

**Figure 3:**
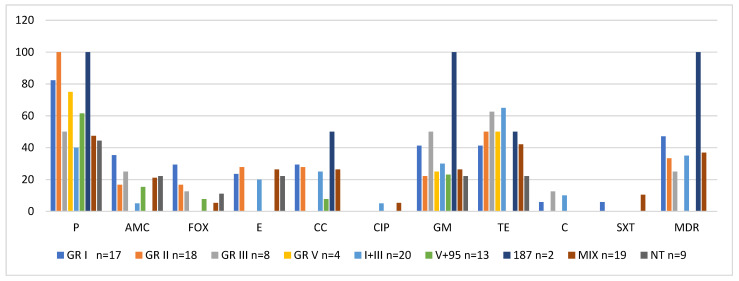
Antibiotic resistance of 110 oral *S. aureus* strains versus their sensitivity to phages from lytic groups. P—penicillin; AMC—amoxicillin/clavulanic acid; FOX—cefoxitin; E—erythromycin; CC—clindamycin; CIP—ciprofloxacin; GM—gentamicin; TE—tetracycline; SXT—sulfamethoxazole/trimethoprim; MDR—multidrug resistance; MIX—mixed group NT—nontypeable.

Strains with the highest antibiotic resistance belonged to phage lytic group I; the vast majority of strains from this group were resistant to penicillin (82.3%), whereas a smaller proportion showed resistance to gentamycin, tetracycline, amoxicillin/clavulanic acid, cefoxitin, clindamycin, erythromycin, cotrimoxazole, and chloramphenicol. The only antibiotic to which all phage group I isolates were sensitive was ciprofloxacin. Phage group I also contained the highest proportion of multidrug-resistant strains (47.1%) (Figure 3, Table 2).

The only antibiotic to which all strains showing sensitivity to group II phages (3A, 3C, 55, 71) were resistant was penicillin; additionally, 50% of strains from this group showed resistance to tetracycline. Most isolates were sensitive to other antibiotics (amoxicillin/clavulanic acid, cefoxitin, clindamycin, erythromycin, and gentamycin), and all strains showed sensitivity to ciprofloxacin, chloramphenicol, and cotrimoxazole (Figure 3, Table 2).

Strains typeable with phages from group V and group V plus phage 95 turned out to be the most antibiotic-sensitive. All isolates from phage lytic group V were sensitive to 7 out of 10 examined antibiotics, except penicillin, tetracycline, and gentamycin. Only some individual strains typeable with group V phages plus phage 95 showed sensitivity to the examined antibiotics. Notably, none of the isolates were identified as a multidrug-resistant strain (MDR). The antibiotic resistance of isolates from other phage lytic groups is presented in Table 2.

Aside from resistance to beta-lactams, which is a typical feature of MRSA, methicillin-resistant strains typeable with the group I phages were also resistant to tetracycline, gentamycin, erythromycin, and clindamycin. MRSA typeable with phages 3C/71 from group II showed inducible macrolide and lincosamide resistance to (MLSB_i_) (Table 1).

### 2.4. Carriage of Toxin Genes

Both MSSA and MRSA strains typeable with the group II phages 3A, 3C, and 71 carried exfoliative toxin A gene (eta). MSSA susceptible to phage 55 harbored genes of exfoliative toxins A and B (eta/etb). MRSA typeable with phages 80 and 81 carried Panton–Valentine leucocidin genes (lukS/lukF-PV); aside from the eta gene, the strains typeable with phages 3C/71 harbored enterotoxin gene cluster (egc). MRSA strains typeable with group III phage 75 carried the gene for toxic shock syndrome toxin (tst) (Table 1).

### 2.5. Statistical Analysis

All calculations were performed with Statistica 10 package (StatSoft, Tulsa, OK, USA) with the threshold of statistical significance set at *p*-value ≤ 0.05. The significance of differences in the percentages of antibiotic-resistant isolates was verified with Pearson’s chi-squared test or Fisher’s exact test.

## 3. Discussion

Despite growing knowledge of the oral microbiome, the role of *S. aureus* in the oral diseases and the risk of staphylococcal infection spread from this reservoir are still not completely understood [20]. Even less is known about the sensitivity of oral *S. aureus* isolates to bacteriophages. 

The phages analyzed in this study, introduced by the Central Public Health Laboratory at Colindale (London), is the international set used for phage typing of *S. aureus* in 35 countries worldwide [21]. Although phage typing has been recognized for years as a method for epidemiological analysis, the applicability of this technique was recently limited by the emergence of multiple nontypeable *S. aureus* strains [22]. Therefore, we decided to analyze the lytic activity of bacteriophages from various groups against oral *S. aureus* isolates in the context of their antibiotic resistance. Nearly all isolates included in this study turned out to be sensitive to phages from the basic set, and only 9 out of 110 (8.2%) strains were shown to be nontypeable. Our findings differ considerably from the results reported by other authors, according to whom the percentages of nontypeable strains were markedly higher, from 30% up to 60% [22,23,24]. Presumably, this discrepancy may be associated with the origin of the strains, as the staphylococci analyzed in previous studies were isolated from other anatomical regions than the oral cavity.

The relationship between the origin of *S. aureus* isolates and sensitivity thereof to selected phages is a well-established phenomenon. Hospital strains were shown to be primarily typeable with group I and III phages, whereas the majority of those isolated in a community setting were typeable with group II phages [25,26]. The results of the present study suggest that phages from mixed groups, I+III and a mixed group containing phages belonging to at least three various lytic groups, showed the highest lytic activity against oral *S. aureus*. A similar tendency could also be observed in recent studies, which demonstrated the highest lytic activity of the mixed-group phages [23,24].

MRSA and MSSA differ in terms of their phage sensitivity. For years, MSSA strains have been shown to be predominantly sensitive to group II phages, whereas group III sensitivity seems to be a characteristic feature of MRSA [25,26,27]. However, it is now considered that a large proportion of MRSA isolates are nontypeable with phages from the basic set. This finding was not confirmed in our present study, as all but one oral MRSA isolate (91.7%) were highly typeable. Our MRSA strains were primarily sensitive to 80/81 phages from group I. Nosocomial outbreaks caused by phage type 80/81 *S. aureus* were previously reported in many countries, including Canada, Australia, United States, United Kingdom, Norway, and Denmark. These strains typically emerged at neonatal and surgical wards and were highly infectious [28]. While the outbreaks caused by phage type 80/81 strains are reported less often nowadays, Manal et al. found a considerable proportion of type 80/81 among CA-MRSA [23]. Our findings are consistent with these results, as the presence of type IV SCC*mec* in phage type 80/81 strains suggested CA-MRSA. Unlike *S. aureus* isolated from other infections, oral MRSA strains were sensitive to phages 3C/71 from group II. Previously, the sensitivity was considered a characteristic feature of MSSA associated with skin infections [29]. However, Manal et al. identified similar MRSA strains [23]. 

The phage sensitivity of *S. aureus* has been changing over time. These changes were usually associated with the introduction of novel antibiotics and the resultant selection of strains with the new pattern of phage sensitivity. For many years, most *S. aureus* isolated in a hospital setting have been sensitive to group I and III phages [27]. It was postulated that the primary reason behind the spread of these strains in a hospital environment is their high antibiotic resistance. Indeed, in our present study, the cluster of isolates typeable with the group I phages contained the highest proportion of strains resistant to antibiotics, such as penicillin, gentamycin, tetracycline, amoxicillin/clavulanic acid, cefoxitin, clindamycin, erythromycin, cotrimoxazole and chloramphenicol. Meanwhile, the sensitivity to group V and group V with phage 95 was associated with a high susceptibility to most antibiotics, except penicillin and tetracycline. Thus, it may be hypothesized that the survival and spread of oral *S. aureus* are not associated with their antibiotic resistance but with high colonization potential. Phage type 95 strains were shown to be strong and stable colonizers, and this mechanism is plausibly involved in the case of oral *S. aureus* as well [30]. Our findings are consistent with the results published by Kareieve et al., according to whom phage type 95 MSSA strains were also sensitive to antibiotics [26].

Bacteriophages are considered the main risk factor for acquiring the elements of genetic virulence by *S. aureus*. They also participate in the transmission of pathogenicity islands and are the primary carrier of chromosomal and extrachromosomal genes during horizontal gene transfer (HGT) [31]. Phages can transfer genes of various staphylococcal virulence factors, including Panton–Valentine leucocidin, staphylokinase, enterotoxins, chemotaxis inhibitory proteins, and exfoliative toxins [32,33]. This may explain the association between the sensitivity of *S. aureus* to some phages and their pathogenicity. *S. aureus* of phage group II isolated from skin infections/abscesses often produce exfoliative toxins, leading to Ritter’s disease [34]. In our present study, the isolates harboring exfoliative toxin genes, whether MSSA or MRSA, turned out to be typeable with group II phages. Importantly, epidemic CA-MRSA strains sensitive to phages 80/81 were demonstrated to carry genes for Panton–Valentine leucocidin. PVL is a cytotoxin with strong pro-inflammatory properties, involved in the etiopathogenesis of necrotic pneumonia and constituting a health threat for patients colonized by PVL-positive *S. aureus* [35]. 

## 4. Materials and Methods

### 4.1. Isolation of Oral S. Aureus Strains

The study was carried out on 110 strains of *S. aureus* isolated from oral samples of patients with symptoms of oral infections in 2016–2018. Oral swabs were analyzed at the Laboratory of Oral Microbiology of the Medical University of Gdansk in accordance with routine laboratory procedures. The analyzed staphylococcal strains were not specifically isolated for this research; they were part of the diagnostic laboratory procedure and no humans were involved in the experiments.

The materials were streaked onto blood agar and Chapman agar (bioMerieux) and were incubated 18–24 h at 37 °C. After incubation, colonies with typical staphylococcal morphology (size, shape, or pigmentation) were identified biochemically with the API ID32 STAPH test (bioMerieux), and uncertain identification was additionally confirmed by the MALDI-TOF MS method.

After final identification, the isolates were stored at −80 °C in Trypticase Soy Broth (Becton Dickinson, Franklin Lakes, NJ, USA) supplemented with 20% glycerol for further use. 

### 4.2. Lytic Activity of Bacteriophages of Basic International Set

Phage typing was performed by the standard method using the phages of the Basic International Set and, additionally, 88, 89, 187 phages at RTD [36]. The RTD (Routine Test Dilution) is the highest dilution just failing to give confluent lysis. The International Basic Set of typing phages consist of 23 phages in five lytic groups: I lytic group with phages 29, 52, 52A, 79, 80, 81; II lytic group with phages 3A, 3C, 55, 71; III lytic group with phages 6, 4E, 47, 53, 54, 75, 77, 83A, 84, 85; V lytic group with phages 94, 96; and miscellaneous group with 95 and other phages.

Phages were propagated on the homologous propagating strains, which were first subcultured on a blood agar plate, and a colony was picked for use. The overnight nutrient broth culture of the propagating strain was added to the new broth medium to give a final dilution of 1/100. Phages were then added to give a final dilution equivalent to 1 × RTD/mL, and the volume of lysate added to the broth medium depended on the phage titer. The mixtures of propagating strain and phage were incubated at 37 °C preferably with shaking for 6 h. After incubation, the lysate was centrifuged and the supernatant pipetted off and titrated.

To determine the phage titer, tenfold dilutions of the phage (from supernatant) were made in nutrient broth and a 0.02 mL drop of each dilution was spotted on a lawn of the propagating strain. Plates containing 0.7% nutrient agar supplemented with 400 µg/mL of calcium ions were used in this stage of typing. After overnight incubation at 30 °C, the phage titer and its RTD were calculated. Phage reactions were read for lysis with a 10-x hand lens and reactions were recorded. The RTD was defined as the highest dilution of a phage that just fails to give confluent lysis on the homologous propagating strain. 

Isolates of *S. aureus* were subcultured in nutrient broth and incubated at 37 °C to give an inoculum of about 5 × 10^7^ CFU/mL. Nutrient agar plates with 400 µg/mL of calcium ions were flooded with bacterial culture and allowed to dry open at room temperature. After the plates dried, they were spotted with the phages of the Basic International Set at RTD and additional phages. A small volume of phage suspensions at RTD (0.01 mL/ drop) was applied in a standard arrangement and the plates were allowed to dry. After overnight incubation at 30 °C, phage reactions were read for lysis with a 10-x hand lens and reactions were recorded as follows irrespective of the size of the phage plaques: on a scale of ±(up to 19 plaques), +(20–49 plaques) as weak lysis, ++(50 or more plaques, confluent lysis) as strong lysis, -(no reaction) as negative. The patterns of phage reactions were created from the numbers of the phages that lysed the investigated strain of *S. aureus.*

### 4.3. Antibiotic Resistance Testing

Antibiotic susceptibility was determined by the disk diffusion method according to the EUCAST [37]. The following antibiotics were tested: penicillin G 1 unit, cefoxitin 30 µg, amoxicillin/clavulanic acid 20/10 µg, clindamycin 2 µg, erythromycin 15 µg, ciprofloxacin 5 µg, gentamicin 10 µg, tetracycline 30 µg, trimethoprim/sulfamethoxazole 1,25/23, 75 µg, chloramphenicol 30 µg (Bio-Rad, Marnes la Coquette, France and Oxoid, Basingstoke, UK). Multidrug resistance (MDR) was defined as a resistance to three or more classes of non-β-lactam antimicrobials.

### 4.4. Methicillin-Resistance and Staphylococcal Cassette Chromosome Mec (SCCmec) Detection

The Genomic Micro AX Staphylococcus Gravity kit (A&A BIOTECHNOLOGY, Gdynia, Poland) was used to isolate genomic DNA from bacteria by gravity in accordance with the manufacturer’s instructions.

Screening of methicillin resistance was determined by a cefoxitin disc in accordance with EUCAST recommendations and confirmed by the detection of the PBP2a protein (OXOID PBP2 ‘Latex Agglutination Test Kit). Methicillin resistance was verified by the detection of the *mec*A and *mec*C genes [38,39]. Methicillin-susceptible *S. aureus* ATCC25923 and methicillin-resistant *S. aureus* ATCC43300 were used as control strains. 

For *mec*-positive strains, five major staphylococcal chromosomal cassettes *mec* (I–V) were determined as described by Oliveira et al. [40] and by Milheiriço et al. [41]. The SCC*mec* type was determined on the basis of the band pattern profiles obtained. 

### 4.5. Detection of Major Staphylococcal Toxin Genes

Detection of major toxin genes for isolated *S. aureus* was performed on the basis of the enterotoxins (*sea*, *seb*, *sec*, *sed*, *see*), additional enterotoxins (*seg*, *seh*, *sei*, *sej*, *sec*, *sel*, *sem*, *sen*, *seo*, *seu*), exfoliative toxins (*eta*, *etb*), toxic shock syndrome toxin-1 (*tst*), and Panton–Valentine leukocidin genes (*lukS / F*-PV) [35,42,43]. 

## 5. Conclusions

To summarize, this study showed that both methicillin-sensitive and methicillin-resistant oral *S. aureus* isolates were generally susceptible to phages from the international bacteriophage set. The highest lytic activity showed that phages 79 and 52A from lytic group I. *S. aureus* strains sensitive to phages group I were usually resistant to penicillin and susceptible to ciprofloxacin, whereas the strains typeable with group V or group V with the 95 phage were susceptible to most antibiotics. Epidemic CA-MRSA strains of phage type 80/81 carried Panton–Valentine leucocidin genes, while *S. aureus* typeable with group II phages harbored exfoliative toxin genes (*eta*/*etb*), which determined their high pathogenic potential. Considering the high sensitivity of *S. aureus* to analyzed phages and the promising results of phage therapies reported by other authors, phage cocktails or phage-antibiotic combinations may potentially find application in both the prevention and eradication of staphylococcal infections.

## Figures and Tables

**Table 1:** Characteristics of methicillin-resistant oral *S. aureus* (MRSA).

Phage Group	Phage Susceptible	Antibiotic Resistance	*mec*A/*mec*C Genes	Type SCCmec	Toxin Genes
I	81 ^++^	P, AMC, FOX, E, CC	*mec*A	V	*lukS-PV/lukF-PV seb, sek*
I	80 ^++^	P, AMC, FOX, TE, SXT	*mec*A	V	*lukS-PV/lukF-PV seb, sek*
I	52A ^++^	P, AMC, FOX,	*mec*A	IV	*sec*
I	81 ^++^	P, AMC, FOX	*mec*A	IV	none
I	81 ^++^	P, AMC, FOX, TE, GM	*mec*A	IV	*egc*
II	3C ^++^	P, AMC, FOX,	*mec*A	IV	none
II	3C ^++^, 71 ^++^	P, AMC, FOX, E, CC, TE	*mec*A	IV	*egc, eta*
II	3C ^++^, 71 ^++^	P, AMC, FOX, E, CC, TE	*mec*A	IV	*egc*
III	75 ^+^	P, AMC, FOX, TE	*mec*A	V	*tst, egc*
V and 95	95 ^++^, 96 ^++^	P, AMC, FOX,	*mec*A	IV	*egc*
I, III, V and 95	29 ^++^, 52 ^++^, 52A ^++^, 79 ^++^, 80 ^++,^, 81 ^++^, 6 ^++^, 42E ^++^, 53 ^++^, 54 ^++^, 75 ^++^, 77 ^++^, 83A ^++^, 85 ^++^, 88 ^++^, 89 ^++^, 95 ^++^, 96 ^++^	P, AMC, FOX, GM	*mec*A	IV	*sec*
NT	NT	P, AMC, FOX	*mec*A	V	*sec*

Abbreviations: NT—nontypeable; P—penicillin; AMC—amoxicillin/clavulanic acid; FOX—cefoxitin; E—erythromycin; CC—clindamycin; CIP—ciprofloxacin; GM—gentamicin; TE—tetracycline; C—chloramphenicol; SXT—trimethoprim–sulfamethoxazole. *Pvl*—Panton–Valentine leucocidin genes; *eta*—exfoliative toxin gene; *tst*—toxic shock syndrome toxin-1 gene; *seb*, *sec*, *sek*—enterotoxins genes; *egc* (*seg, sei, sem, sen, seo, seu*)—enterotoxins cluster genes; ^++^, ^+^—intensity of lytic reaction.

**Table 2:** Sensitivity to specific lytic phages of MSSA and MRSA 110 oral *S. aureus* strains and their antibiotic resistance. P—penicillin; AMC—amoxicillin/clavulanic acid; FOX—cefoxitin; E—erythromycin; CC—clindamycin; CIP—ciprofloxacin; GM—gentamicin; TE—tetracycline; SXT—sulfamethoxazole/trimethoprim.

Phage Lytic Groups																											
**Group I Group II Group III Group V**																											
**Specific phages**																											
	**29**	**52**	**52A**	**79**	**80**	**81**	**3A**	**3C**	**55**	**71**	**6**	**42E**	**47**	**53**	**54**	**75**	**77**	**83A**	**84**	**85**	**94**	**96**	**95**	**187**	**88**	**89**	
Ant. **Number of phage-sensitive MSSA strains** Total (%)																											
	6	7	30	36	17	6	5	7	17	2	17	9	4	2	7	7	0	3	13	14	27	23	24	2	5	17	307 (100)
**P**	5	4	10	17	10	3	5	7	17	2	5	4	2	2	4	3		2	3	6	16	12	12	2	3	4	160 (52.1)
**AMC**	1	1	1	4	3			1	1	1	1	2		1	1	2			1	2	4	2	2			1	32 (10.4)
**FOX**																											
**E**	1	1	10	3	3		1	2	2	1	2	1			2	2			4	3	4	2	3		1	4	52 (16.9)
**CC**	1	2	9	6		1	1	1	2		3					1			2	2	5	2	4	1	1	4	48 (15.6)
**CIP**				1					1		1										1	1				1	6 (2.0)
**TE**	2	4	13	17	11	5	3	3	8	1	9	8	1		5	3		3	7	5	11	4	5	1	2	9	140 (45.6)
**GM**	1	2	9	8	7	4	1		5		5	1	1		3	2		2	2	2	9	6	6	2	1	5	84 (27.4)
**C**			2		1						2	1			1				1	2							10 (3.3)
**SXT**				2					1		1								1		1	1	1				8 (2.6)
**Phage lytic groups**																											
**Group I Group II Group III Group V**																											
**Specific phages**																											
	**29**	**52**	**52A**	**79**	**80**	**81**	**3A**	**3C**	**55**	**71**	**6**	**42E**	**47**	**53**	**54**	**75**	**77**	**83A**	**84**	**85**	**94**	**96**	**95**	**187**	**88**	**89**	
Ant. **Number of phage-sensitive MRSA strains** Total (%)																											
	1	1	2	1	2	4	0	3	0	2	1	1	0	1	1	2	1	1	0	1	0	2	2	0	1	1	31 (100)
**P**	1	1	2	1	2	4		3		2	1	1		1	1	2	1	1		1		2	2		1	1	31 (100)
**AMC**	1	1	2	1	2	4		3		2	1	1		1	1	2	1	1		1		2	2		1	1	31 (100)
**FOX**	1	1	2	1	2	4		3		2	1	1		1	1	2	1	1		1		2	2		1	1	31 (100)
**E**						1		2		2																	5 (16.1)
**CC**						1		2		2																	5 (16.1)
**CIP**																											
**TE**					1	1		2		2						1											7 (22.3)
**GM**	1	1	1	1	1	2					1	1		1	1	1	1	1		1		1	1		1	1	19 (61.3)
**C**																											
**SXT**					1																						1 (3.2)

## Data Availability

Not applicable.
